# Reversal of Experimental Liver Damage after Transplantation of Stem-Derived Cells Detected by FTIR Spectroscopy

**DOI:** 10.1155/2017/4585169

**Published:** 2017-12-28

**Authors:** Danna Ye, Philip Heraud, Rangsun Parnpai, Tong Li

**Affiliations:** ^1^Department of Reproductive Medical Center, The First Affiliated Hospital of Wenzhou Medical University, Wenzhou, China; ^2^Embryo Technology and Stem Cell Research Center, School of Biotechnology, Suranaree University of Technology, Nakhon Ratchasima, Thailand; ^3^Department of Anatomy and Developmental Biology, Monash University, Melbourne, VIC, Australia; ^4^Center for Biospectroscopy, School of Chemistry, Monash University, Melbourne, VIC, Australia; ^5^School of Ophthalmology and Optometry, Eye Hospital, Wenzhou Medical University and Key Laboratory of Vision Science, Ministry of Health, Wenzhou, China

## Abstract

The transplantation of autologous BM-MSCs holds great potential for treating end-stage liver diseases. The aim of this study was to compare the efficiency of transplanted rBM-MSCs and rBM-MSC-derived differentiated stem cells (rBM-MSC-DSCs) for suppression of dimethylnitrosamine-injured liver damage in rat model. Synchrotron radiation Fourier-transform infrared (SR-FTIR) microspectroscopy was applied to investigate changes in the macromolecular composition. Transplantation of rBM-MSC-DSCs into liver-injured rats restored their serum albumin level and significantly suppressed transaminase activity as well as the morphological manifestations of liver disease. The regenerative effects of rBM-MSC-DSCs were corroborated unequivocally by the phenotypic difference analysis between liver tissues revealed by infrared spectroscopy. Spectroscopic changes in the spectral region from 1190–970 cm^−1^ (bands with absorbance maxima at 1150 cm^−1^, 1081 cm^−1^, and 1026 cm^−1^) indicated decreased levels of carbohydrates, in rBM-MSC-DSC-transplanted livers, compared with untreated and rBM-MSC--transplanted animals. Principal component analysis (PCA) of spectra acquired from liver tissue could readily discriminate rBM-MSC-DSC-transplanted animals from the untreated and rBM-MSC-transplanted animals. We conclude that the transplantation of rBM-MSC-DSCs effectively treats liver disease in rats and SR-FTIR microspectroscopy provides important insights into the fundamental biochemical alterations induced by the stem-derived cell transplantation, including an objective “signature” of the regenerative effects of stem cell therapy upon liver injury.

## 1. Introduction

Liver damage often leads to liver fibrosis which sometimes progresses to liver cirrhosis [1]. Liver transplantation is one of the most effective treatments for severe liver-associated diseases such as cirrhosis. However, due to the shortage of donated organs and the growing list of patients in need of such intervention, transplantation is often not a viable option [2]. Current studies suggest that hepatocyte transplantation may develop into a feasible alternative to whole-organ transplantation; however, the efficiency of isolation of sufficient transplantable hepatocytes is very low and is restricted by the small number of marginal donor organs allocated for this purpose [3–5]. Hence, novel cell sources are required to deliver hepatocytes of adequate quality for clinical use. Most of the recent studies concentrate on stem cells of extrahepatic origin, as a potential derivation source for producing hepatocytes, because of their ready availability and unrestricted potential to propagate and differentiate [6–9].

The preeminent candidate stem cells for therapy for injured livers are mesenchymal stem cells (MSCs), which possess multipotentiality ability, and *in vitro* have the potential to differentiate into hepatocyte-like cells [10, 11]. Moreover, studies have shown that rat or human mesenchymal stem cells can differentiate into hepatocyte-like cells when transplanted into rat liver [12–14]. Recently, transplantation of rat bone marrow-derived mesenchymal stem cells (rBM-MSCs) has been shown to protect the rat liver from chemically induced liver fibrosis and improves some hepatic functions [15–17]; however, their effectiveness was reduced by the limitation of characterization of the cells that were transplanted. Even though the evidence that bone marrow-derived cells suppress fibrosis in mice has been shown [18, 19], it remains controversial which type(s) of cells among those derived from the bone marrow show the most potent suppressive effect on fibrosis.

FTIR microspectroscopy is a powerful technique, which has been widely used in biophysical research, and has been proven to provide sensitive and precise measurement of biochemical changes in a diverse range of biological cells and tissue [20]. For example, FTIR imaging analysis is becoming a valuable analytic method in brain research showing the ability to detect tumour formation [21] and very early changes associated with autoimmune encephalomyelitis [21]. Wang et al. used FTIR microspectroscopy to study the compositional changes in inflammatory cardiomyopathy, and the results demonstrate chemical difference between the inflammatory responses in the mouse model, providing insight into why the disease can be self-limiting in some cases while fatal in others [22]. Recently, synchrotron infrared microspectroscopy has been used for the early detection of liver fibrosis [23]. In addition, FTIR microspectroscopy also can be used to distinguish between stem cells and their differentiated cells of human [24–26] and murine stem cells [27–30]. The infrared spectroscopic approach provides structural information about macromolecules, such as proteins, nucleic acids, carbohydrates, and lipids, allowing detection, identification, and quantification of changes in these cellular components associated with changes in biological state. These spectroscopic approaches to phenotypic characterization of disease progression are facilitated typically by sophisticated multivariate modeling and classification methods [31].

In this study, we aimed to compare the efficiency of rBM-MSCs with differentiated stem cells derived from BM-MSCs to suppress dimethylnitrosamine-induced liver injury in rats, by comparing a range of conventional histological and blood analyses with synchrotron radiation Fourier transform infrared (SR-FTIR) microspectroscopy, which was applied to investigate possible biochemical molecular alteration of the liver tissue after the transplantation of cells.

## 2. Materials and Methods

### 2.1. Cell Culture

All animal care and surgical interventions were undertaken in strict accordance with the approval of the Suranaree University of Technology Laboratory Animals Ethics committee. rBM-MSCs were isolated from 8-week-old female Wistar rats and cultured as described previously [32]. rBM-MSCs at passage five were used in this study. Differentiated stem cell differentiation from rBM-MSCs and characterization were performed as described by our previous reports [33]. In brief, rBM-MSCs were serum-deprived for 2 days in Iscove's Modified Dulbecco's Medium (IMDM) supplemented with 10 ng/ml basic fibroblast growth factor (bFGF) and 20 ng/ml epidermal growth factor (EGF). Then, rBM-MSCs were cultured in IMDM supplemented with 20 ng/ml hepatocyte growth factor (HGF), 10 ng/ml bFGF, and 4.9 mmol/ml nicotinamide for 7 days. Finally, the cells were treated with IMDM supplemented with 10 mmol/ml ITS (insulin, transferrin, and selenious acid), 1 mmol/ml dexamethasone, and 20 ng/ml oncostatin M for 14 days. The media were changed twice weekly. Before transplantation, the differentiated cells were characterized by liver-specific proteins and gene expression and liver function determination. Our latest reports show that rBM-MSC-DSCs were able to chronologically expressed liver-specific proteins like AFP, ALB, CK18, HNF1*α*, and HNF3*β*. rBM-MSC-DSCs expressed liver-specific genes such as CYP2B1, ALB, and CXCR4 [33].

### 2.2. DMN-Induced Rat Liver Injury Model

Female Wistar rats were bred and maintained in an air-conditioned animal house with specific pathogen-free conditions and were subjected to a 12 : 12-hours daylight/darkness and allowed unlimited access to food and water. Liver damage was induced by dimethylnitrosamine (DMN) in 3-week-old rats, weighing between 180 and 200 g. DMN administration was as follows: on day 0, rats were injected intraperitoneally at a dose of 100 *μ*L DMN (diluted 1 : 100 with 0.15 mol/L sterile saline) per 100 g body weight. The same volume of sterile saline alone was used as control. The injections were given on three consecutive days of each week for 4 weeks. Cirrhosis was determined by killing three rats per week for histopathology. A total of 28 rats were used in this study. All reagents were purchased from Sigma-Aldrich, unless otherwise indicated.

### 2.3. Cell Transplantation

Untreated rBM-MSCs at passage five, as well as rBM-MSC-DSCs, were prepared for cell transplantation. To monitor the transplanted cells, those cells were stained using the PKH Fluorescent Cell Linker kit as the instructions described. PKH26-derived fluorescence was observed using a fluorescence microscope (Olympus, Tokyo, Japan). Cell transplantation was performed as described by our previous reports [33]. The DMN-treated rats were randomly divided into three groups after 4 weeks DMN treatment: (a) the DMN/rBM-MSCs group, injected intravenously a dose of rBM-MSCs of 1 × 10^6^ cells per rat, *n* = 5; (b) the DMN/ rBM-MSC-DSCs group, injected intravenously a dose of differentiated stem cells of 1 × 10^6^ cells per rat, *n* = 5; and (c) DMN/saline group, which were injected with 1 ml of saline, only *n* = 5. DMN untreated rats were regarded as the normal group. On day 28, venous blood was collected for assessment of liver function. Albumin (ALB), aspartate aminotransferase (AST), and alanine transaminase (ALT) levels were assessed using conventional laboratory methods [16]. All rats were killed and the liver tissue was harvested for further analysis. Histopathology of the liver was conducted using our previously described protocol [33]. All values are presented as mean ± S.E.M, the data were performed for statistical significance using ANOVA and followed by Tukey HSD post hoc correction, with *P* < 0.05 considered statistically significant.

### 2.4. Synchrotron Infrared Microspectroscopy (SR-FTIR)

High spatial resolution infrared spectral maps were collected at the infrared microspectroscopy beamline (2BM1B) at the Australian Synchrotron, Melbourne, Australia. SR-FTIR spectra were acquired using a Hyperion 2000 FTIR microscope (Bruker Optik GmbH, Ettlingen, Germany) with a narrow-band mercury cadmium telluride (MCT) detector coupled to a Bruker Vertex 80 V FTIR spectrometer, which was connected to an IR beamline at the Australian Synchrotron. The sample was mapped through the focused beam using an X-Y step size of 4 *μ*m with a 4 *μ*m × 4 *μ*m aperture in the microscope focal plane with a spectral resolution of 8 cm^−1^ with 64 interferograms coadded. All spectral acquisition and control functions of the microscope were performed through Bruker Opus version 6.5.

### 2.5. Data Analysis

Spectra from all liver samples were extracted using the CytoSpecTM (Cytospec Inc., Boston MA, USA) spectroscopic software after performing a quality test to assess the appropriate sample thickness, rejecting the spectra with maximum absorbance less than 0.2 or greater than 0.8 absorbance units over the spectrum range of 3100–970 cm^−1^. Spectra extracted using Cytospec were subsequently converted into a galactic format by a macro converter in OPUS6.5 software in preparation for multivariate data analysis.

Representative spectra from all groups were processed and compared following multivariate analysis. Following quality selection, 1020 spectra were then selected randomly from each experimental group with an equal number of spectra from each rat. 340 spectra acquired from each animal liver section were further reduced to 85 spectra by taking the arithmetic mean of groups of 4 spectra, selected at random. 255 spectra from each experimental group were further randomly selected to 85 by taking the arithmetic mean of groups of 3 spectra. Prior to multivariate analyses or classification, the data was preprocessed by performing the second derivative using the Savitzky-Golay algorithm with 9 smoothing points and normalization using the extended multiplicative signal correction (EMSC). The Unscrambler 9.7 software (Camo Software AS, Oslo, Norway) was used for multivariate data analysis.

## 3. Results

### 3.1. Changes in the Liver Tissue in the Liver-Damaged Rat Model

**Figure 1:**
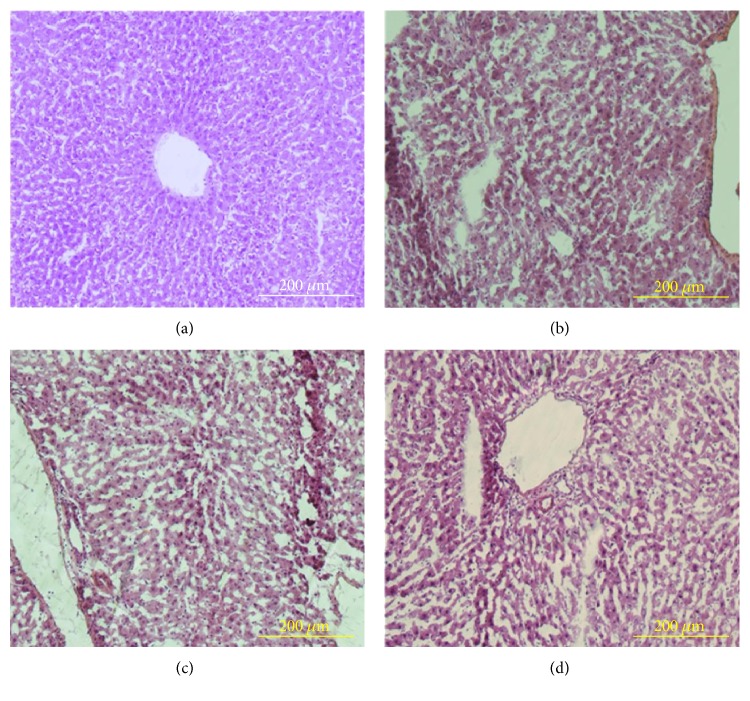
Representative liver tissues stained with hematoxylin and eosin. Normal liver (a), 1-week injection of DMN (b), 2 weeks injection of DNM (c), and 4 weeks injection of DMN (d). Original magnification, 100x.

Routine H&E staining was employed to characterize representative liver sections at 1 week, 2 weeks, and 4 weeks following DMN injection and liver sections from rats not injected with DMN that served as controls, as shown in Figure 1. The liver tissue from control rats not exposed to DMN showed the normal histological appearance of hepatocytes, that is, they were polyhedral with eosinophilic cytoplasm and a central nucleus (Figure 1(a)). However, after 1 week following DMN injection, necrosis areas appeared (Figure 1(b)). Specifically, after 2 weeks of DMN injection, the large areas of necrosis were found (Figure 1(c)). At the fourth week of injection, the alteration of the liver structure was even more evident, with more hemorrhagic necrosis and disruption of tissue architecture (Figure 1(d)).

### 3.2. Tracing of Transplanted Cells in the DNM-Injured Liver

**Figure 2:**
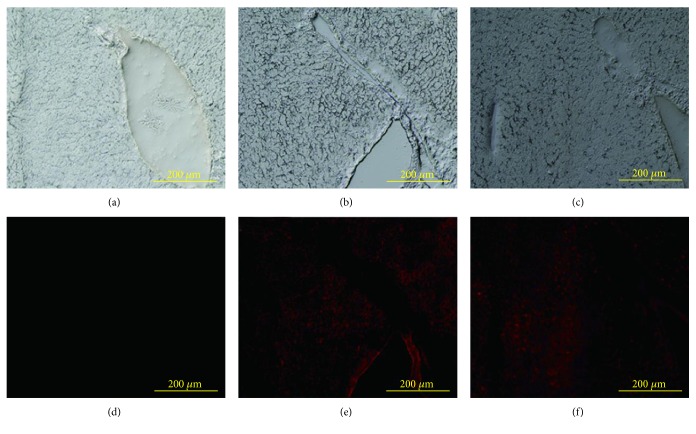
Engraftment of PKH-stained rBM-MSCs and rBM-MSC-DSCs in DMN -injured rat livers. Nontransplanted liver from DMN-damaged rats was used as the control (a and d). rBM-MSCs (b and e) and rBM-MSC-DSCs (c and f) were transplanted into DMN-damaged rats, and 4 weeks later liver sections were observed using fluorescence microscopy. The upper panel (a, b, and c) and lower panel (d, e, and f) picture are bright-field and fluorescence images, respectively, with PKH-stained cells within the tissue fluorescing red. Original magnification, 200x.

PKH26-stained rBM-MSCs and rBM-MSC-DSCs were transplanted into DMN-damaged rats to examine what cell type was effective for the engraftment in the liver.  Figure 2 shows the fluorescence microscopy images of liver tissues from the animals transplanted with PKH26-stained rBM-MSCs and rBM-MSC-derived hepatocytes. The PKH26-stained cells were easily detected in the liver by fluorescence microscopy. The transplanted cells were located in the blood vessels, the sinusoid, and the liver lobules. This result suggested that the transplanted cells entered the sinusoid and liver parenchymal tissue.

### 3.3. Recovery of Albumin Production by Stem Cell Transplantation

**Figure 3:**
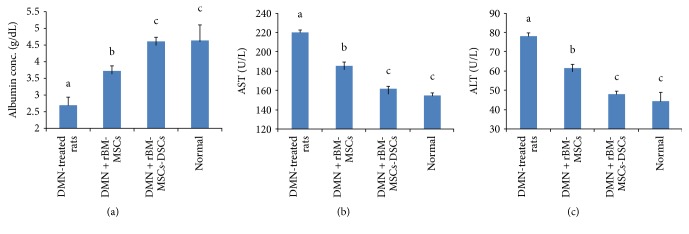
Biochemical analysis of blood sera. (a) Concentration of albumin in blood serum of rats. (b) Concentration of aspartate aminotransferase (AST) in blood serum of rats. (c) Concentration of alanine transaminase (ALT) in blood serum of rats. Bars with different letter superscripts are different statistically (*P* < 0.05).

The normal level of rat serum albumin was 4.64 ± 0.46 g/dL, while the DMN treatment rat serum albumin was 2.71 ± 0.25 g/dL, which was significantly lower than the normal level (*P* < 0.05, Figure 3(a)). Liver-injured rats recovered serum albumin levels but these were still lower than that of the normal level following transplantation of rBM-MSCs. In contrast, the transplantation of rBM-MSC-DSCs into liver-damaged rats restored the serum albumin close to the normal level. Although it is not clear whether it was the transplanted rBM-MSC-DSCs that produced albumin or whether DMN-damaged liver regenerated in response to the transplanted cells, nevertheless, transplantation of the differentiated stem cells effectively led to restored albumin production after the transplantation.

### 3.4. Suppression of Liver Inflammation by Stem Cell Transplantation

The AST and ALT levels in the serum of normal rats not exposed to DMN were 155.1 ± 2.21 and 45.4 ± 4.79 U/L, respectively. As shown in Figures 3(b) and 3(c), the serum AST and ALT levels in the DMN-treated rats were 220.2 ± 2.43 and 76.1 ± 1.82 U/L, respectively, which were significantly higher than the normal level (*P* < 0.05). The transplantation of rBM-MSC-DSCs significantly suppressed the serum AST and ALT levels to the normal levels in the DNM-injured rats. The transplantation of rBM-MSCs suppressed the serum AST and ALT level in the DNM-injured rats to some extent, but this was still higher than normal levels and the difference was statistically significant. In summary, it appeared that transplantation of the differentiated stem cells effectively suppressed liver inflammation caused by the DMN treatment and was significantly more effective than transplantation of undifferentiated rBM-MSCs.

**Figure 4:**
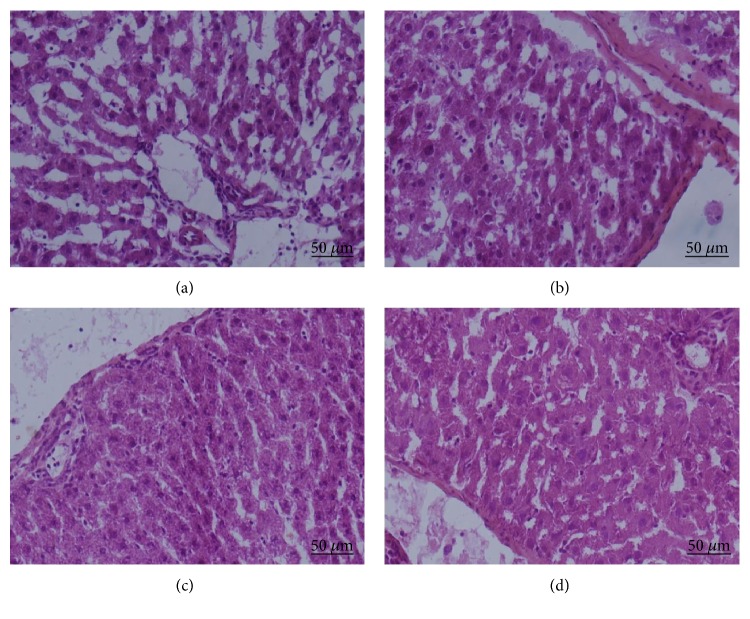
Hematoxylin and Eosin staining of liver sections from DNM-injured rats that received cell transplant. (a) DNM-injured rats did not receive cell transplant showed hemorrhagic necrotic and disruption of tissue architecture. (b) DNM-injured rat received rBM-MSC transplant showed some changes of necrosis areas and the tissue architecture. (c) DNM-injured rat received rBM-MSC-DSC transplant showed significant changes of necrosis areas and the tissue architecture. (d) Normal liver. Original magnification, 200x.

In addition to the serum protein assays, the effects of rBM-MSCs and rBM-MSC-DSCs on a DMN-injured liver were evaluated by histopathologic examination of the liver sections by H&E staining. The control group (Figure 4(a)) exhibited the hemorrhagic necrotic and disruption of tissue architecture. Some changes of necrosis areas and the tissue architecture in the liver sections were observed in transplantation of rBM-MSCs group (Figure 4(b)). Differences were more marked in the liver tissue architecture following transplantation of rBM-MSC-DSCs. Hemorrhagic necrosis was rarely observed in these tissues and tissue architecture and appeared to be similar to that of normal (control) rats (Figure 4(c)).

### 3.5. Synchrotron Radiation Fourier-Transform Infrared (SR-FTIR) Microspectroscopy Investigation of Liver Tissue

**Figure 5:**
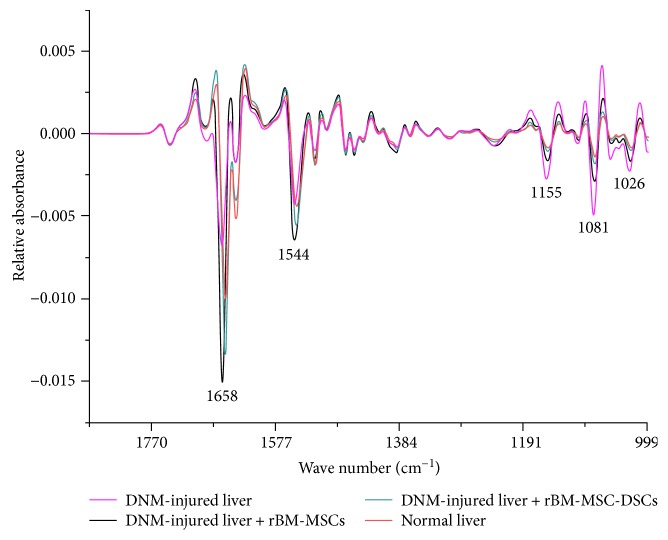
Average second derivative FTIR spectra from 1800 to 950 cm^−1^.

SR-FTIR microspectroscopy was applied to investigate any macromolecular phenotypic changes in the liver tissue after transplantation of rBM-MSCs and rBM-MSC-DSCs, compared to DMN-injured rats that did not receive cellular transplants and control rats not subjected to the DMN treatment.  Figure 5 shows average second derivative IR spectra from 1800 to 950 cm^−1^ in each group. The average spectra showed differences in bands near 1658 cm^−1^ (amide I mode from proteins), 1544 cm^−1^ (amide II mode from proteins), as well as IR absorbance bands with maxima at 1155 cm^−1^, 1081 cm^−1^, and 1026 cm^−1^ which were assigned to glycogen and other carbohydrates [34]. The intensities of *α*-helix (1658 cm^−1^) and amide II (1544 cm^−1^) were highest in liver tissue after rBM-MSCs injection, followed by the liver tissue injected with rBM-MSC-DSCs compared to the normal liver tissue, indicating different protein content in each tissue type. It was also shown that bands from carbohydrates at 1155 cm^−1^, 1081 cm^−1^, 1026 cm^−1^ were highest in DMN-injured liver tissue (control) and followed by liver tissue from rats transplanted with rBM-MSCs and lowest in the liver tissue from rats transplanted rBM-MSC-DSCs. The profile of these bands in liver tissue from rBM-MSC-DSCs transplanted rats was very similar to that of normal liver tissue not exposed to DMN (Figure 5). Elevation of glycogen levels in liver tissue following solvent-induced damage compared to healthy tissue is well known [23]. Our results showing increased absorbance in C-O stretching bands from carbohydrates in damaged tissue compared to normal controls is consistent with these observations. Moreover, the decrease in the intensity of these bands after stem cell treatment, particularly by rBM-MSC-DSCs, indicated a decrease in glycogen levels indicative of amelioration of the solvent-induced liver damage by these treatments.

**Figure 6:**
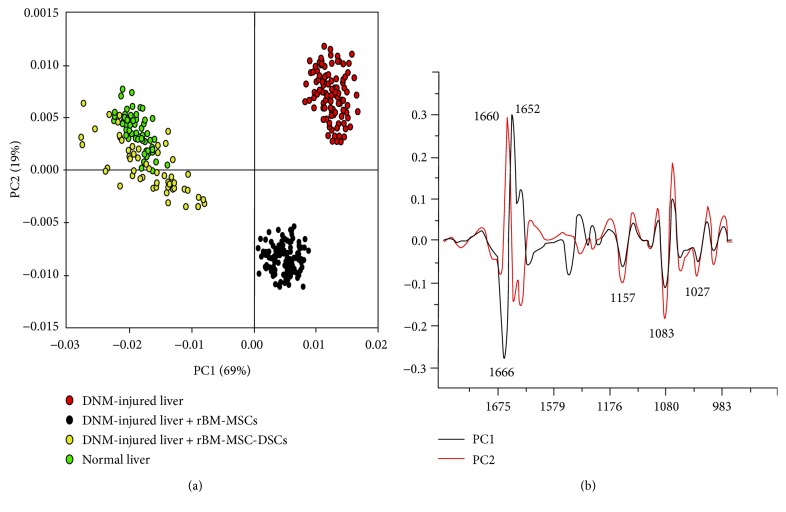
Principal component analysis (PCA) score plots (a) and loading plots (b). Spectra were processed to the 2nd derivative over the spectral range between 1770–1500 cm^−1^ and 1190–970 cm^−1^ prior to PCA. Average spectra for DNM-injured liver (*n* = 85), DNM-injured liver + rBM-MSCs (*n* = 85), DNM-injured liver + rBM-MSC-DSCs (*n* = 85), and normal liver (*n* = 85) from *N* = 3 rats for each experiment. Each point on the score plot represents a mean spectrum, with each calculated from 12 spectra. Principal component 1 (PC1) is a vector in the direction of the greatest variance in the data set (explaining 69% of total variance), and principal component 2 (PC2) is in the direction of the next major source of variance (explaining 19% of total variance) that is independent to variance explained by PC1. The loadings plots show which spectral bands were most responsible for the clustering observed in the scores plot.

Given that the spectroscopic dataset was inherently multivariate in nature, principal component analysis (PCA) was applied to assess relative changes in band profiles across the population of spectra acquired from liver tissue samples. PCA of the SR-FTIR spectra was performed in the 1770–1500 cm^−1^ and 1190–970 cm^−1^ spectral region which is associated with protein and glycogen absorbance. In the score plot (Figure 6(a)), PC1 was explained by 69% of total variance and PC2 was explained by 19% of total variance. The loading plots (Figure 6(b)) shows which spectral bands were most responsible for the clustering observed in the score plot. The PCA score plot showed that the spectra extracted from the control group, rBM-MSCs injection group, rBM-MSC-DSCs injection group, and normal liver were clustered separately along PC1 (Figure 6(a)). Spectra from normal liver tissue were colocalised on the score plot with spectra from the tissue of rats exposed to DMN and subsequently transplanted with rBM-MSC-DSCs (Figure 6(a)), corroborating the similarity between average spectra from these classes (Figure 5). In contrast, spectra from the DMN-exposed control liver tissue and rBM-MSC-injected liver tissue clustered separately from spectra from untreated normal liver tissue and rBM-MSC-DSCs transplanted tissue along the PC1 axis (Figure 5(a)). Further, spectra from DMN-exposed control liver tissue and rBM-MSCs injection liver tissue clustered separately along the PC2 axis (Figure 6(a)) in the PC1 versus PC2 score plot. Loadings plots (Figure 6(b)) were examined to determine spectra changes that were most influential on the clustering patterns observed in the score plot. PC1 loadings showed prominent negative loadings at 1157 cm^−1^, 1083 cm^−1^, and 1027 cm^−1^ that were inversely correlated with a strong positive loading 1652 cm^−1^. These loading indicated that DMN-treated control and rBM-MSCs transplanted liver tissue spectra had stronger absorbance for bands assigned to glycogen (maxima at 1157 cm^−1^, 1083 cm^−1^, and 1027 cm^−1^) and lower absorbance for proteins (amide I band at 1652 cm^−1^) compared with normal untreated and rBM-MSC-DSC-transplanted samples, corroborating the differences observed in the average spectra (Figure 5). PC2 loadings were similar to PC1 loadings indicating that liver tissue from DMN-treated animals receiving no cellular transplants had the highest glycogen and lowest protein levels compared to DMN animals that were transplanted with rBM-MSCs, again corroborating the differences observed between the average spectra (Figure 5).

## 4. Discussion

In our previous study and in this study, we showed the effectiveness of transplanting rBM-MSC-DSCs to treat liver damage in an experimental animal model using dimethylnitrosamine, which was consistent with previous study where carbon tetrachloride was used as a hepatotoxic agent [16]. We were able to refine knowledge in this area showing that hepatic differentiation of rBM-MSCs pretransplantation enhanced the engraftment of new cells in the recipient's liver and produced clear therapeutic benefits compared with transplantation of undifferentiated rBM-MSCs in DMN-injured rats. Specifically, we found that the transplantation of rBM-MSC-DSCs significantly reduced the serum transaminase levels in DMN-injured rats and appeared to be more effective for the suppression of liver inflammation compared with transplantation of the undifferentiated stem cells. Previous studies have reported that hepatocyte growth factor (HGF) upregulated C-X-C chemokine receptor type 4 (CXCR4), which is the chemokine receptor for stromal cell-derived factor-1 (SDF-1), in human hematopoietic stem cells [35]. SDF-1 is expressed in the liver bile duct epithelium and the secretion is increased by the inflammation. The injected rBM-MSC-DSCs have been proven to express CXCR4 in our previous report. We hypothesize that CXCR4 might be upregulated in rBM-MSC-DSC-induced HGF and the engraftment in injured livers could be enhanced by the interaction with SDF-1. In support of this view, a previous study has shown that CCl_4_-injured hepatocytes stimulated HGF secretion in the cocultured BM-MSCs [16]. HGF is well known to suppress hepatocyte death and liver fibrosis [28]. The results suggest that when transplanted into a DMN-injured rat, engrafted rBM-MSC-DSCs might secrete HGF in the liver and suppress the inflammation.

However, our results were contrary to previous work reporting that undifferentiated rBM-MSCs were the most effective for suppression of liver fibrosis compared to rBM-MSC-DSCs [36]. It is still not clear why and how the undifferentiated rBM-MSCs most effectively suppressed liver fibrosis in this previous work, or why the opposite was the case in our study; however, the different results of our current study may have been due to the different transplantation approach we employed. In our study, we chose intravenous injection rather than intrasplenic injection used in the previous work, which is an easy and convenient way of cell delivery, being less invasive and traumatic to the recipient [36]. Furthermore, intravenous injection of transplanted cells has shown to have more efficient migration to the target areas, because the injured target organ may express specific receptors or ligands to facilitate trafficking of transplanted cells [36, 37]. Other issues need to be taken into account, including the age of rat used in experimental liver disease, the protocol of induced liver disease, and the hepatic differentiation protocol. In our study, a 3-week-old rat was used for transplantation compared to an 8-week-old rat used in Hardjo's report. The rat at the early age seems to recover more efficiency than the old. In addition, we could not compare the degree of liver fibrosis in our study to Hardjor's study because the different protocols were used to induce liver fibrosis. The degree of liver fibrosis is an important issue that affects the results of cell transplantation. Lastly, the hepatic differentiation method we used was different to Hardjor's study. The method we used has reported more efficiency in hepatic differentiation in our previous report [31].

A unique aspect of the current study was the use of SR-FTIR microspectroscopy to obtain insights into biochemical changes in the tissue occurring as result of liver damage and as a result of transplantation of rBM-MSCs and derived cells. The spectral phenotypic “signature” of each experimental group was shown to be significantly different and was associated with differences in protein and glycogen levels. The lowest protein levels found in the liver tissues of DMN-treated animals not receiving transplants is consistent with findings that DMN destroys proteins and inhibits further synthesis protein [38]. The higher levels of protein absorbance found in rBM-MSCs and rBM-MSC-DSCs transplantation groups compared to DMN-treated animals not receiving transplants suggested the resumption of protein synthesis after cell transplantation. However, the mechanism of how the transplanted cells could cause this is still unclear. It was also shown that glycogen levels were lower in rBM-MSC-DSCs treated liver than that in the control group and rBM-MSCs treated group. The liver plays a major role in carbohydrate metabolism, with DMN causing loss of liver function, particularly the destruction of glycogenolysis, which leads to accumulation of glycogen in the liver [38]. Glycogen was observed to have the highest level in the control group, indicating the accumulation of glycogen in the livers of these animals [23]. However, glycogen levels in animals receiving cellular transplantation, especially in those transplanted with rBM-MSC-DSCs, suggested a decrease of glycogen deposition in the liver, which resulted from the resumption of carbohydrate metabolism in the livers of these animals after cell transplantation. In the rBM-MSC-DSCs transplantation group, the spectral profile of glycogen bands was found to be similar to that of normal liver tissue, indicating similar biochemical composition and confirming that rBM-MSC-DSCs effectively treated and reversed the liver damage induced by DMN.

## 5. Conclusion

The transplantation of rBM-MSC-DSCs effectively treated the liver injury in rats. Transplantation of these cells restored serum albumin level and significantly suppressed transaminase activity and liver disease. This is a promising technique for autologous transplantation in humans with liver injury. The changes of cellular composition revealed by infrared spectroscopy indicated that rBM-MSC-DSCs caused the resumption of protein synthesis and carbohydrate metabolism in the liver after transplantation leading to the recovery of normal function.
